# 796. Burden of Healthcare-associated (HA) Respiratory Syncytial Virus (RSV) in Hospitalized Adults

**DOI:** 10.1093/ofid/ofab466.992

**Published:** 2021-12-04

**Authors:** Alexandra C Hill-Ricciuti, Edward E Walsh, William G Greendyke, Angela Barrett, Luis Alba, Angela Branche, Ann Falsey, Matthew R Phillips, Yoonyoung Choi, Lyn Finelli, Lisa Saiman

**Affiliations:** 1 Columbia University Irving Medical Center, Torrington, Connecticut; 2 University of Rochester, Rochester, New York; 3 Rochester General Hospital, Rochester, New York; 4 Merck & Co., Inc., North Wales, PA; 5 Merck, Philadelphia, Pennsylvania

## Abstract

**Background:**

Little is known about the burden of HA-RSV in hospitalized adults. We assessed risk factors and clinical outcomes in hospitalized adults diagnosed with HA-RSV.

**Methods:**

A retrospective case-control study was performed from 2017-2020 in two academic hospital systems. HA-RSV cases were >18 years of age, hospitalized >4 days, and developed new or worsening respiratory signs and symptoms that prompted clinicians to test for respiratory pathogens; cases were RSV-positive by PCR assays. Two community-onset (CO) RSV-positive controls (admitted with >2 acute respiratory symptoms), were matched to each HA-RSV case by age, sex, and RSV season. We compared risk factors and outcomes in cases vs. controls. We assessed escalation of respiratory support in HA-RSV cases, defined as new or increased respiratory support from Day -2 to Day +4 of their RSV-positive test. Exact conditional logistic regression compared outcomes of HA-RSV vs. CO-RSV subjects, adjusting for demographic and clinical characteristics.

**Results:**

84 cases and 160 controls (both median 64 years) were included; 87% had ≥1 comorbidity. HA-RSV cases were hospitalized for a median of 10 (IQR: 5-17) days prior to their RSV-positive test. CO-RSV controls were more likely to have pulmonary comorbidities than HA-RSV cases (46% vs. 31%, p=0.02). 38% of HA-RSV vs. 15% of CO-RSV subjects were hospitalized ≥15 days after their RSV-positive test (p=0.047). 14% of HA-RSV and 6% of CO-RSV subjects died (p=0.25). Among patients who survived, HA-RSV cases were more likely discharged to a skilled nursing or rehabilitation facility than CO-RSV controls (46% vs. 17%, p=0.04). Of the 44 HA-RSV cases assessed thus far, 25% required escalation of respiratory support; none required initiation of mechanical ventilation.

**Conclusion:**

HA-RSV was associated with increased morbidity and increased health care resource use during and after hospitalization. RSV vaccines could prevent CO- and HA-RSV infections in adults.

**Disclosures:**

**Edward E. Walsh, MD**, **GSK** (Advisor or Review Panel member)**Janssen** (Grant/Research Support)**Merck** (Grant/Research Support)**Merck** (Grant/Research Support) **William G. Greendyke, MD**, **Merck** (Grant/Research Support) **Angela Branche, MD**, **Merck Dohme and Sharpe** (Grant/Research Support) **Ann Falsey, MD**, **BioFire Diagnostics** (Grant/Research Support)**Janssen** (Grant/Research Support)**Merck** (Grant/Research Support)**Novavax** (Other Financial or Material Support, DSMB member)**Pfizer** (Grant/Research Support) **Matthew R. Phillips, MPH**, **Merck & Co., Inc.** (Employee, Shareholder) **Yoonyoung Choi, PhD, MS, RPh**, **Merck** (Employee) **Lyn Finelli, DrPH, MS**, **Merck** (Employee) **Lisa Saiman, MD, MPH**, **Merck** (Grant/Research Support, Research Grant or Support)**Merk Co., Inc** (Grant/Research Support, Advisor or Review Panel member)

